# Characterization of Potential Molecular Markers in Lac Insect *Kerria lacca* (Kerr) Responsible for Lac Production

**DOI:** 10.3390/insects13060545

**Published:** 2022-06-14

**Authors:** Nawaz Haider Bashir, Weiwei Wang, Xiaofei Ling, Jinwen Zhang, Qin Lu, Rui He, Hang Chen

**Affiliations:** 1 Institute of Highland Forest Science, Chinese Academy of Forestry, Kunming 650224, China; nawazhaider@caf.ac.cn (N.H.B.); sk121www@caf.ac.cn (W.W.); lingxf0305@foxmail.com (X.L.); jinwen@caf.ac.cn (J.Z.); lqin@caf.ac.cn (Q.L.); herui61@aliyun.com (R.H.); 2 The Key Laboratory of Cultivating and Utilization of Resources Insects, State Forestry Administration, Kunming 650224, China

**Keywords:** lac insect, single-nucleotide polymorphisms (SNPs), insertions and deletions (InDels), variants, annotation, candidate genes

## Abstract

**Simple Summary:**

Lac insect *Kerria lacca* (Hemiptera, Kerriidae) is widely used to produce lac in tropical and subtropical areas. This species is genomically understudied and there is no information present on single-nucleotide polymorphisms and insertions and deletions of lac insects. This study involves distribution and characterization of molecular markers in *K. lacca*. Nucleotide substitution frequency and transition to transversion ratio were highest at the late adult stage. The maximum numbers of SNPs and InDels were distributed on chromosome 8 and 1, respectively. Most variants at low frequencies were detected in InDels compared to SNPs. Gene function analysis showed several gene variations, and candidate SNPs that were involved in lac synthesis. This work paves the way to determining further mechanisms involved in increased lac production.

**Abstract:**

*Kerria lacca* (Kerr) is an important lac insect extensively used in industrial products in the form of resin, wax and dye. The scarce knowledge on molecular markers for *K. lacca* is a barrier in elucidating genetic information. Our study identified a total of 16,921 single-nucleotide polymorphisms (SNPs), and 6231 insertions and deletions (InDels)—of which, intergenic variation accounted for 41.22% and 56.30%, and exonic variation accounted for 39.10% and 17.46%, of SNPs and InDels, respectively. Observation of SNPs suggested that nucleotide substitution frequency and transition to transversion (Ts/Tv) ratio were highest at the late adult stage, 3.97, compared to at the other stages, with a genome-wide Ts/Tv ratio of 2.95. The maximum number of SNPs, 2853 (16.86%), was identified in chromosome 8, while the lowest, 1126 (6.65%), was identified in chromosome 7. The maximum and minimum numbers of InDels were located on chromosome 1 and 7, with 834 (13.38%) and 519 (8.33%), respectively. Annotation showed that highest numbers of exonic and intergenic SNPs were present at the late adult stage, whereas the maximum number of InDels was found at the larval stage. On the basis of gene function, 47 gene variations were screened and 23 candidate genes were identified in associations with lac production. Concluding work will enhance knowledge on molecular markers to facilitate an increase in lac production in *K. lacca* as well as other lac insects.

## 1. Introduction

Lac insects are known as solitary animals with significant ability to produce natural resin [1]. The natural product produced is called lac, which is composed of three main components—lac resin, wax, and dye [2]. These components have important applications in cosmetics, food, textiles, pharmaceutical industries, surface coating, and electrical industries [3]. Lac is widely used in several industries due to properties including moisture resistance, non-toxic, strong adhesiveness, tasteless, good insulation, and smooth film texture [4].

Lac resins are a combination of lactides and lactones made of polyterpene esters and hydroxyl fatty acids [5]. In cytoplasm, mevalonate (MVA) is a biosynthetic pathway in the formation of polyterpene esters [6,7]. The pathway involves conversion of acetyl-CoA into mevalonic acids through the action of hydroxylmethylglutaryl-CoA synthase and reductase. Further, mevalonic acid is converted into sesquiterpene acids through the action of terpene synthases (TPS) followed by conversion of sesquiterpene acids to sesquiterpene acid esters [8]. Hydroxyl fatty acids, the second components, are formed through fatty acid synthesis by passing through different steps catalyzed by fatty acid desaturases (FAD), elongases of very long chain fatty acids (ELO), and fatty acid synthetases (FAS). Another important constituent of lac resins is aleuritic acid (−9, 10, 16-erythro aleuritic acid), which is produced from acetyl CoA, and fatty acid synthases [9].

**Figure 1:**
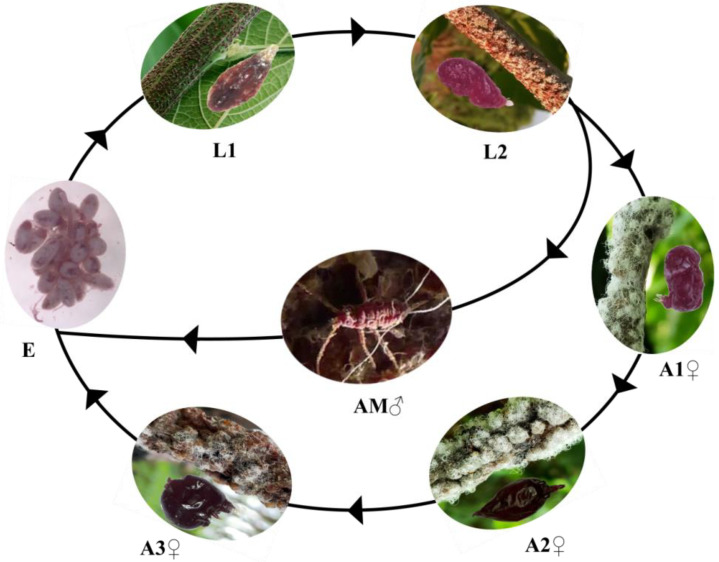
Life cycle of lac insects: females and males are morphologically different to each other. Showing eggs (E), early instar larvae (L1), late instar larvae (L2), early adult (A1), mid adult (A2), late adult (A3), and adult male (AM).

Lac insects are members of the genus *Kerria* (Hemiptera, Kerriidae) [10], with 29 species worldwide [11], of which *K. lacca* species is most widely cultivated for commercial lac production [12]. *Kerria lacca* is well distributed in China, India, Pakistan, Azerbaijan, Bangladesh, Georgia, Guyana, Malaysia, Myanmar, Nepal, Sri Lanka, and Thailand, and reported on more than one hundred host plants [13,14]. Females and males of *K. lacca* showed sexual dimorphism [15], and both produce and secrete remarkably different proportions of lac during their life cycle [4]. The life cycle of female lac insects is approximately 6–8 months and involves eggs, nymph instars and the adult stages (Figure 1) [16]. Female larvae and males produce small amounts of lac, whereas adult females (in the early, mid and late adult stages) are capable of secreting huge amounts of lac [17].

Knowledge on molecular markers is always a primary need for genomic research [18]. In terms of DNA markers, single-nucleotide polymorphisms (SNPs) and insertions and deletions (InDels) are the most valuable markers [19]. These markers (SNPs and InDels) are associated with protein-coding genes and their pertinent translated regions [20], and are expedited to understand their link to gene function [21]. Several studies were carried out on lac insects in the terms of ecology, biology, genetic diversity, microbial diversity, host plants, molecular phylogeny, biogeography, and transcriptome [1,4,14,22,23]. However, *K. lacca* is also genomically understudied insect, and there are no data available on SNPs and InDels of lac insects. Therefore, the present study involves the use of *K. lacca* for genome-wide identification of DNA variants through an Illumina NovaSeq. Potential variants (SNPs and InDels) were studied to develop and enlist lac synthesis genes to predict and observe its effect using genomic location. The linked variants will be further used to facilitate genotype studies and for marker-assisted selection for lac synthesis.

## 2. Materials and Methods

### 2.1. Insect Material

*Kerria lacca* was reared on host plant *Schleichera oleosa* in the Research Station of Yuanjiang, Institute of Highland Forest Science, Yunnan, China (102°00′46″ E, 23°36′11″ N). Female lac insects at the larvae (*n* = 100), early adult (*n* = 100), mid adult (*n* = 100) and late adult (*n* = 100) stages were collected. Immediately, all samples were put in liquid nitrogen, then transferred and stored at −80 °C. The samples were used for genomic DNA and variation studies as follows.

### 2.2. Sequencing and Variant Identification

Total genomic DNA was extracted from 1 g/sample (3 biological replication per stage) using an insect DNA Extraction Kit, Zymo Research (Irvine, CA, USA), following the manufacturer’s instructions. DNA quality was assessed using a NanoDrop (ND2000) spectrophotometer (Thermo Fisher Scientific, Waltham, MA, USA), and extracted DNA was stored at −20 °C for future use and library preparation. The whole-genome library was prepared using a library preparation kit Nextera Flex Library Prep Kit (Illumina, San Diego, CA, USA) according to the manufacturer’s instructions, with an average insert size of 150 bp, and was sequenced on the Illumina NovaSeq 6000 at Benagen Technology Co. Ltd. (Wuhan, China). The raw data were quality controlled using FastQC (version 0.20.1) [24] and clean reads were aligned to the reference genome of the *K. lacca* species with default parameters. SAMtools (version 1.11) was used to delete the duplicate reads to discover and filtrate variants [25] to sort, remove duplicated reads and merge the bam alignment results of each sample. GATK (version 4.2.0.0) [26] software was used to detect variation on data to obtain high-quality SNP and InDel information, with these parameters (SOR > 4.0; QD < 2.0; MQ < 40.0; FS > 60.0; DP < 10.0; ReadPosRankSum < −8.0).

### 2.3. Annotation of Single-Nucleotide Polymorphisms and Insertion/Deletion Polymorphism

SnpEff (version 4.3) was used to determine the putative effect of DNA variants [27]. The SnpEff tool was used to annotate a variant call format (VCF) file containing SNPs and InDels using default parameters, and variants were classified as genic and intergenic according to their genomic location. Variants in intergenic and intron regions are classified as modifier impact. Variants in coding genic regions can have two effects: a moderate to low impact or a high impact. Moderate- to low-impact variants (e.g., synonymous variants) are thought to be mainly innocuous or unlikely to affect protein behavior, but a non-disruptive variant that may affect protein efficacy is termed as moderate impact (e.g., inframe deletion and non-synonymous variant). High-impact variants (e.g., frameshift variant and stop gained) may cause protein truncation or loss of function [27,28].

### 2.4. Identification of Lac Production-Related Genes with Linked Variants

Based on the lac secretion-minimum (larvae) and lac secretion-active (early, mid, and late adult) stages, diverse variations (SNPs and InDels) were analyzed. We selected differentially expressed genes (DEGs) during the lac production-minimum and -maximum stages including fatty acid metabolism, fatty acid biosynthesis, fatty acid elongation, and terpenoid backbone biosynthesis. We listed lac-related genes as those identified with high expression during the lac production-maximum stage. The protein sequences of DEGs were used as queries to identify genes with related functions. We explored the association with SNPs and InDels variants from selected lac-related genes. The genes linked to variants were classified into four categories: fatty acid metabolism genes, fatty acid biosynthesis genes, fatty acid elongation-related genes, and terpenoid backbone biosynthesis genes. The expression profiles of selected SNPs were indicated as heatmaps through TBtools software (version 0.665) [29].

## 3. Results

### 3.1. SNP and InDel Variant Identification

**Figure 2:**
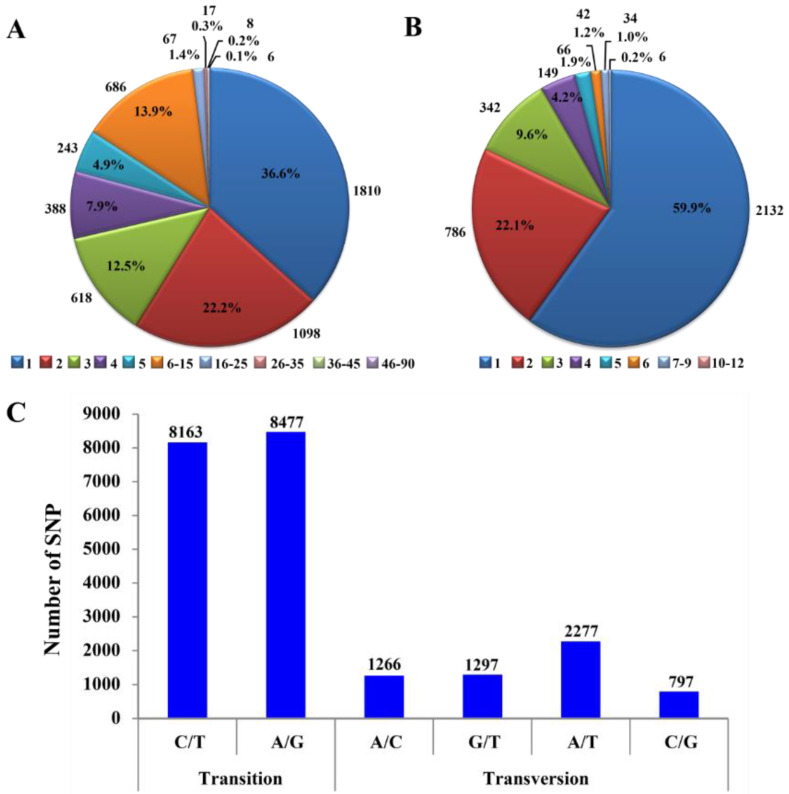
Characterization of single-nucleotide polymorphism (SNP) and insertion and deletion (InDel) markers: (**A**) number of SNPs in a single unigene; (**B**) number of InDels in a single unigene, and (**C**) classification of SNPs including number of SNPs.

We studied the effects of SNPs and InDels on gene functions and their distribution. We detected a total of 16,921 single-nucleotide polymorphism (SNP) sites, with a total number of transition (Ts) and transversion (Tv) alterations of 74.7% and 25.3%, respectively. Of these SNPs, 13.72% were synonymous, while 12.34% were non-synonymous mutants. In addition, among all the SNP calling unigenes, approximately 36.6% (1810 SNPs) have a single SNP marker, and 22.2% (1098 SNPs) and 12.5% (618 SNPs) have double and triple SNPs, respectively. Almost 26.7% (1317 SNPs) have 4–15 SNP markers and one unigene contains the highest number of SNP markers, 86 (Figure 2A). In the region-wise distribution of SNPs, 41.22% (4663) were observed in intergenic regions and 39.10% (4423) variants in exon regions. The variant numbers in 3′UTR and 5′UTR were 14.15% (1601) and 5.12% (579), respectively. Only 47 (0.42%) of variants were found in the splice site regions. The majority of variants (60.49%) have a modifying effect on the genome, comprising 5′UTR, 3′UTR, and intergenic variants (Table 1).

A total of 6231 insertions and deletions (InDels) were recorded in the assembled *K. lacca* transcriptome. Among all the InDel calling unigenes, more than half, 59.9% (2132 InDels), have a single InDel marker, and 22.1% (786 InDels) and 9.6% (342 InDels) have double and triple InDels, respectively. Almost 8.3% (294 InDels) have 4–11 InDel markers, and three unigenes contain the highest number of InDel markers, 12 (Figure 2B). In addition, in the region-wise distribution of InDels, 17.46% (592) variants were observed in exon regions, and 56.30% (1909) variants in intergenic regions. The variant numbers in 3′UTR and 5′UTR were 17.31% (587) and 8.50% (288), respectively. Only 15 (0.44%) variants were found in the splice site regions (Table 1).

### 3.2. SNP Substitutions at Different Stages of Kerria Lacca

On the basis of nucleotide substitutions, SNPs were grouped as transitions (purine to purine and pyrimidine to pyrimidine) and transversions (purine to pyrimidine and pyrimidine to purine). We found 16,640 transitions and 5637 transversions, with a genome-wide transition to transversion ratio (Ts/Tv) of 2.95. Study of SNPs showed that the frequency of nucleotide substitution and Ts/Tv ratio were greater at the late adult stage (3.97), compared to at the mid adult stage (2.74), the early adult stage (2.64) and the larval stage (2.49) (Table 2 and Table S1). Most SNPs were of the A/G (38.05%) type followed by T/C (36.64%) (Figure 2C). Transversion substitutions of SNPs consisted of T/A (10.22%) and A/C (5.68%), followed by G/T (5.82%) and G/C (3.58%) (Figure 2C).

### 3.3. SNP and InDel Chromosomal Distribution

**Figure 3:**
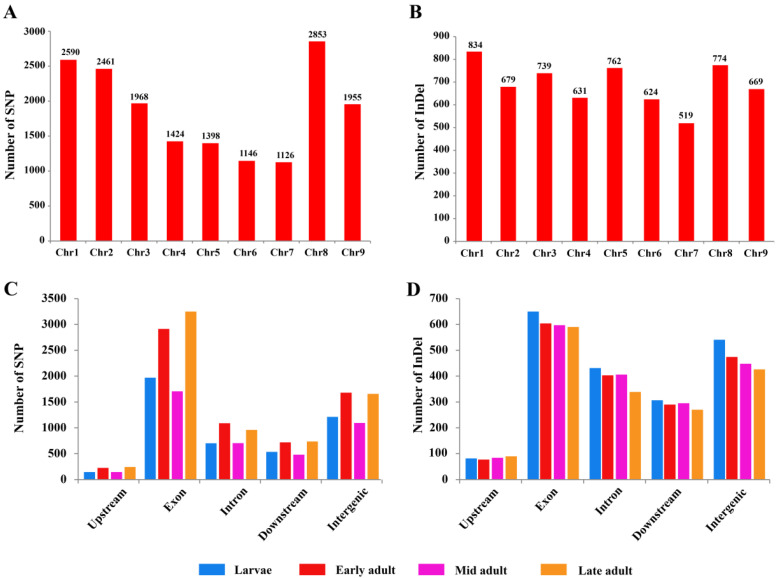
SNP and InDel distribution across *Kerria lacca*: (**A**) chromosomal distribution of SNPs, (**B)** chromosomal distribution of InDels, (**C**) stage-wise SNP annotation, and (**D**) stage-wise InDel annotation.

SNPs and InDels were recognized in *K. lacca* chromosomes. The maximum number of SNPs was detected on chromosome 8 (2853 SNPs) and the minimum on chromosome 7 (1126 SNPs). Chromosome 1 contained 15.31% (2590 SNPs), followed by chromosomes 2, 3, 4, 5, 6, and 9 with 14.54% (2461 SNPs), 11.63% (1968 SNPs), 8.42% (1424 SNPs), 8.26% (1398 SNPs), 6.77% (1146 SNPs) and 11.55% (1955 SNPs), respectively (Figure 3A). The maximum number of InDels was 834 on Chr1, and the minimum was 519 on Chr7. Chromosome 2 contained 10.90% (679 InDels), followed by chromosomes 3, 4, 5, 6, 8, and 9 with 11.86% (739 InDels), 10.13% (631 InDels), 12.23% (762 InDels), 10.01% (624 InDels), 12.42% (774 InDels) and 10.74% (669 InDels), respectively (Figure 3B).

### 3.4. SNP and InDel Annotation

A total of 22,175 SNPs were collected at the larvae, early adult, mid adult and late adult stages of *K. lacca*. SNP annotation revealed that more SNPs were located at upstream sites at the late adult stage (243 SNPs), followed by at the early adult (225 SNPs), mid adult (146 SNPs), and larvae (145 SNPs) stages. The highest number of SNPs were present at exonic sites at the late adult stage (3250 SNPs), the lowest at the mid adult stage (1706 SNPs), and the larvae and early adult stages contained 1970 SNPs and 2914 SNPs, respectively. The early adult stage contained more SNPs at intron sites and the lowest number of SNPs at the larval stage and the mid adult stage. More SNPs were located at downstream sites at the late adult stage (737 SNPs), followed by at the early adult (720 SNPs), larvae (536 SNPs), and mid adult stages (480 SNPs). The highest number of SNPs were present at intergenic sites at the early adult stage (1679 SNPs), and the lowest at the mid adult stage (1095 SNPs), while the larvae and late adult stages contained 1213 SNPs and 1658 SNPs, respectively (Figure 3C and Table S2).

A total of 7404 InDels were collected at the larvae, early adult, mid adult and late adult stages of *K. lacca*. InDel annotation revealed that more InDels were located at upstream sites at the late adult stage (90 InDels), followed by at the mid adult (84 InDels), larvae (82 InDels), and early adult stages (77 InDels). The highest number of InDels were present at exonic sites at the larval stage (650 InDels), and the lowest at the late adult stage (590 InDels), while the early and mid adult stages contained 604 InDels and 597 InDels, respectively. The larval stage contained more InDels at intron sites and the lowest at the late adult stage. More InDels were located at downstream sites at the larval stage (307 InDels), followed by at the mid adult (295 InDels), early adult (290 InDels), and late adult stages (270 InDels). The highest number of InDels were present at intergenic sites at the larval stage (541 InDels), and the lowest at the late adult stage (426 InDels), while the early and mid adult stages contained 474 InDels and 448 InDels, respectively (Figure 3D and Table S3).

### 3.5. Functional Classification Variants

**Figure 4:**
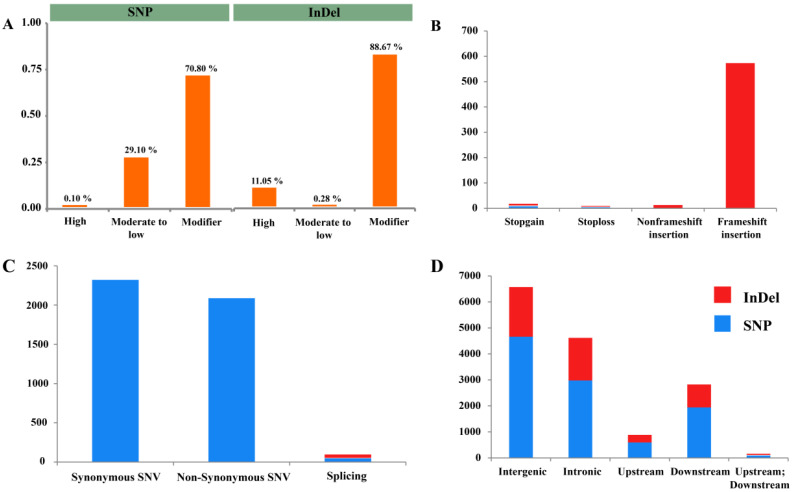
InDel and SNP effects by impact and subclassification: (**A**) number of InDel and SNP effects by impact, (**B**) high impact, (**C)** moderate to low impact, (**D)** modifier impact.

According to genomic position, collectively, 20,092 effects were predicted based on 8498 DNA variants. The effect of one DNA variant can be considered as on multiple gene (e.g., a variant might be upstream one gene while downstream from another gene). SNPs and InDels caused a total of 14,736 and 5356 effects, respectively (Figure 4A). The effects of the variants were classified into three categories: modifier (15,017), moderate to low (4471), and high or strong (604) impact. The SNP effects of the variants were 10,268, 4456, and 12 as modifier, moderate to low and high impact, respectively. The InDel effects of the variants were 4749, 15, and 592 as modifier, moderate to low and high impact, respectively (Figure 4A).

The variants with a high impact had a direct effect on gene function, and we observed a total of 12 SNPs and 592 InDels with this effect. This is mostly caused in SNPs by stop codon gain or loss (Figure 4B) and may have important functional significance. However, in InDels, this is produced by a disturbance in translational reading frame and may lead to improper amino acid sequences and, ultimately, abnormal proteins. A total of 4456 and 15 moderate- to low-impact variants were identified for SNPs and InDels, respectively. In SNPs, moderate impact is due to non-synonymous substitution, which results in a change in one amino acid; and in InDels, this occurs in coding regions (Figure 4C). Low-impact SNPs are largely contained synonymous substitutions without any change in amino acid (Figure 4C). A total of 10,268 SNPs and 4749 InDels were observed with modifier-impact variants, and predicted in non-coding regions (Figure 4D).

### 3.6. Identification of Lac Synthesis Genes

**Figure 5:**
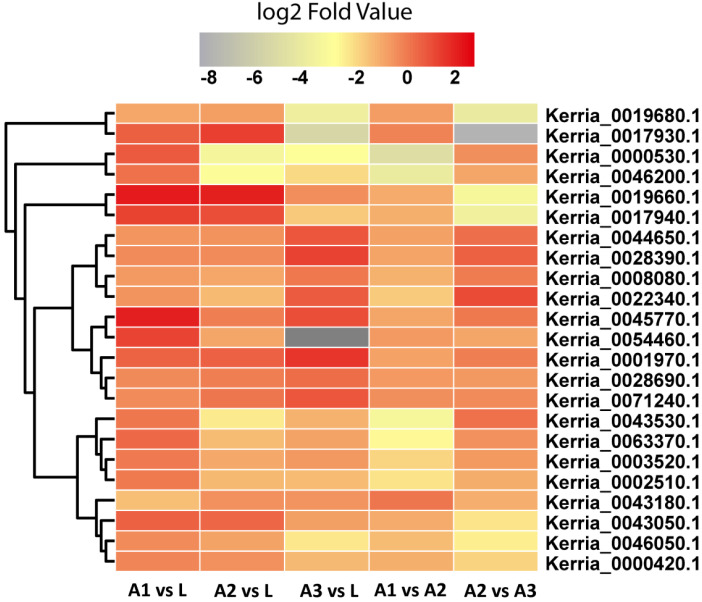
Heatmap of expression profiles of 23 SNPs at different developmental stages of *K. lacca*.

For identification of lac-related genes, we analyzed variations (SNPs and InDels) in the lac secretion-minimum and lac secretion-active stages of the insect. We selected 47 DEGs during the lac production-minimum and -maximum stages determined through gene expression in fatty acid metabolism and terpenoid backbone biosynthesis. From 47 selected lac-related genes, a total of 30 genes were associated with 114 variants (74 SNPs and 40 InDels) (Tables S4 and S5). Of these variants, 44 are located in intergenic regions and 19 variants down and upstream of the genes. Only 40 variants are located inside the genes, including 24 in introns and 16 in exons. Of 30 genes, 20 were associated with both SNPs and InDels, while 13 and 7 variants were specifically associated with SNPs and InDels only, respectively. The 23 lac-related genes with linked variants were categorized into four groups, of which 6 genes were related to terpenoid backbone biosynthesis, 5 to fatty acid metabolism, 6 to both fatty acid biosynthesis and metabolism, and 6 to both fatty acid elongation and metabolism (Table 3). The expression of these SNP variation genes at different developmental stages of *K. lacca* is shown in Figure 5.

## 4. Discussion

A total of 16,921 single-nucleotide polymorphisms (SNPs) and 6231 insertions and deletions (InDels) were obtained in this study, of which the maximum numbers of variations were found at chromosomes number 8 (2853 SNPs) and 1 (834 InDels), whereas the lowest numbers of variations were located at chromosomes number 7 (1126 SNPs and 519 InDels). The frequency of SNPs and the Ts/Tv ratio were higher at the late adult stage, compared to at the larvae, early and mid adult stages, revealing a trend of genomic conservation at different stages during evolution. Transversion substitution and frequency of occurrence of *K. lacca* showed similar trends to that of Asian hornet insect *Vespa velutina* [30] and Pygmy grasshopper *Tetrix japonica* [31]. Single-copy variants are called singleton alleles and two-copy variants are known as doubleton alleles [28]. Most variants in *K. lacca* at low frequencies are detected in InDels (2132 singleton) compared to SNPs (1810 singleton). The highest numbers of SNPs (86) were found in only one Kerria_0015610.1 (carbohydrate metabolism) unigene, which was involved in RGN gluconolactonase. The maximum numbers of InDels 11, 12, 12, and 12 were found in the genes Kerria_0038410.1 (inositol-triphosphate 3-kinase), Kerria_0031000.1 (hypothetical protein), Kerria_0016260.1, and Kerria_0018720.1 (DNA polymerase epsilon subunit 1), respectively.

Based on the lac secretion-maximum (early, mid and late adult) and -minimum (larvae) stages and gene expression level, we screened 23 candidate SNPs possibly involved in lac production. These SNPs were included in terpenoid backbone biosynthesis as decaprenyl-diphosphate synthase, farnesyl pyrophosphate synthase, farnesyl diphosphate synthase, acetyl-CoA acetyltransferase, and hydroxymethylglutaryl-CoA reductase. As sesquiterpene acid is the main component of lac [17], SNPs concerned in terpenoid synthesis may take part in the production of sesquiterpene acids. SNPs were involved in fatty acid metabolism: acyl-CoA dehydrogenase, acyl-coenzyme A oxidase, acyl-CoA delta (11) desaturase, and very long chain fatty acid-elongase; SNPs included in both fatty acid metabolism and fatty acid biosynthesis: fatty acid synthase, long-chain acyl-CoA synthetase, and S-malonyltransferase; SNPs involved in both fatty acid metabolism and fatty acid elongation: elongation-very long chain fatty acids-protein-4, 6, 7, and 3-ketoacyl-CoA thiolase. Hydroxyl fatty acid is also a part of lac [4]; hence, SNPs included in fatty acid synthesis may participate in the production of hydroxyl fatty acids. Wang et al. (2019) found 28 candidate genes that were involved in lac secretion; of 23 candidate SNPs, 9 have a similar function including decaprenyl-diphosphate synthase, acyl-CoA dehydrogenase, acyl-CoA synthetase, and fatty acid synthase.

In the current era, the use of molecular markers plays an important role in understanding and mapping of candidate genes, ecology and genetic diversity [32]. SNP and InDel markers displayed more potential due to high reproducibility, number of alleles per locus and polymorphic nature [32,33,34]. The limitation is the high cost in developing these markers followed by scarce information on genomes in some species including *Kerria lacca*. The presence of SNPs and InDels positively affected the lac-related genes of *K. lacca* to study associated diversity, mapping, and marker-assisted selection. Based on this information, the present study started with DNA variants to search associated lac-related genes with backbone biosynthesis and fatty acid metabolism.

## 5. Conclusions

In summary, we characterized the molecular markers (SNPs and InDels) of *K. lacca* and revealed that there are 3-fold more linked SNPs compared to InDels. Based on the lac secretion-maximum and -minimum stages and gene expression level, few SNPs were involved in lac synthesis. Overall, the presence of significant variations in *K. lacca* genes paves the way to determining further mechanisms involved in increased lac production.

## Figures and Tables

**Table 1:** Region-wise distribution of single-nucleotide polymorphisms (SNPs) and insertion and deletions (InDels) in *Kerria lacca*.

	SNP		InDel	
Type	Count	Ratio	Count	Ratio
3′UTR	1601	14.15%	587	17.31%
5′UTR	579	5.12%	288	8.50%
Exon	4423	39.10%	592	17.46%
Intergenic	4663	41.22%	1909	56.30%
Splice sites	47	0.42%	15	0.44%

**Table 2:** Single-nucleotide polymorphism substitutions in *Kerria lacca*.

Stages	Transitions (Ts)	Transversions (Tv)	Ts/Tv Ratio
Larvae	3275	1314	2.49
Early Adult	4832	1828	2.64
Mid Adult	3041	1110	2.74
Late Adult	5492	1385	3.97

**Table 3:** Lac production-related candidate genes with SNP impacts.

Gene ID	Description	Function	Chr	Variant
Kerria_0043050.1	Decaprenyl-diphosphate synthase	TBS	5	SNP_0043050.1 [A/G]
Kerria_0001970.1	Decaprenyl-diphosphate synthase	TBS	1	SNP_0001980.1 [A/T]
Kerria_0000530.1	Farnesyl pyrophosphate synthase	TBS	1	SNP_0000530.1 [G/A]
Kerria_0046200.1	Farnesyl diphosphate synthase	TBS	5	SNP_0046200.1 [T/G]
Kerria_0043530.1	Acetyl-CoA acetyltransferase	TBS	5	SNP_0043540.1 [A/C]
Kerria_0003520.1	Hydroxymethylglutaryl-CoA reductase	TBS	1	SNP_0003520.1 [T/C]
Kerria_0063370.1	Acyl-CoA dehydrogenase	FAM	9	SNP_0063370.1 [G/A]
Kerria_0044650.1	Acyl-CoA dehydrogenase	FAM	5	SNP_0044650.1 [T/G]
Kerria_0028690.1	Acyl-coenzyme A oxidase	FAM	8	SNP_0028690.1 [T/C]
Kerria_0019680.1	Acyl-CoA delta (11) desaturase	FAM	3	SNP_0019690.1 [C/T]
Kerria_0019660.1	Very long chain fatty acid elongase	FAM	3	SNP_0019660.1 [A/G]
Kerria_0043180.1	Fatty acid synthase	FAB, FAM	5	SNP_0043180.1 [A/G]
Kerria_0046050.1	Fatty acid synthase	FAB, FAM	5	SNP_0046050.1 [A/G]
Kerria_0045770.1	Fatty acid synthase	FAB, FAM	5	SNP_0045780.1 [A/G]
Kerria_0028390.1	Long-chain acyl-CoA synthetase	FAB, FAM	8	SNP_0028390.1 [C/T]
Kerria_0071240.1	Long-chain acyl-CoA synthetase	FAB, FAM	2	SNP_0071240.1 [G/A]
Kerria_0008080.1	S-malonyltransferase	FAB, FAM	1	SNP_0008070.1 [G/A]
Kerria_0017940.1	Elongation-very long chain fatty acids-protein-4	FAE, FAM	3	SNP_0017950.1 [C/T]
Kerria_0017930.1	Elongation-very long chain fatty acids-protein-4	FAE, FAM	3	SNP_0017930.1 [C/T]
Kerria_0000420.1	Elongation-very long chain fatty acids-protein-4	FAE, FAM	1	SNP_0000410.1 [A/C]
Kerria_0022340.1	Elongation-very long chain fatty acids-protein-6	FAE, FAM	3	SNP_0022340.1 [A/G]
Kerria_0054460.1	Elongation-very long chain fatty acids-protein-7	FAE, FAM	4	SNP_0054470.1 [C/T]
Kerria_0002510.1	3-ketoacyl-CoA thiolase	FAE, FAM	1	SNP_0002510.1 [C/T]

Note: FAM, fatty acid metabolism; FAB, fatty acid biosynthesis; FAE, fatty acid elongation; TBS, terpenoid backbone biosynthesis.

## Data Availability

All raw sequencing related to our manuscript is deposited to the public database NCBI under accession number ON687507-ON687529.

## Supplementary Materials

The following supporting information can be downloaded at: https://www.mdpi.com/article/10.3390/insects13060545/s1, Table S1: Summary of SNPs at different stages of *Kerria lacca*; Table S2: SNP annotation at different stages of *Kerria lacca*; Table S3: InDel annotation at different stages of *Kerria lacca*; Table S4: Genes associated with SNP variants; Table S5: Genes associated with InDel variants.
