# Supplementary material for: Characterization of Potential Molecular Markers in Lac Insect Kerria lacca (Kerr) Responsible for Lac Production
Source: Insects. 2022 Jun 14;13(6):545. doi: 10.3390/insects13060545 (PMC9225327; doi:10.3390/insects13060545)
Supplement: Supplementary file 1 [file insects-13-00545-s001.zip › insects-1701410-supplementary.pdf]

Supplementary Information

Characterization of Potential Molecular Markers in Lac Insect *Kerria lacca* (Kerr) Responsible for Lac Production

Nawaz Haider Bashir[1], Weiwei Wang[1], Xiaofei Ling[1], Jinwen Zhang[1], Qin Lu[1], Rui He[1], Hang Chen[1,2*]

[1]Institute of Highland Forest Science, Chinese Academy of Forestry, Kunming, China

[2]The Key Laboratory of Cultivating and Utilization of Resources Insects, State Forestry Administration, Kunming, China

Corresponding author: Hang Chen (stuchen6481@gmail.com)

Table S1: Summary of SNPs at different stages of *Kerria lacca*

Sample (Stages)	Replication	A_G	C_T	Transition	A_C	A_T	C_G	G_T	Transversion	Total
Larvae	1	1776	1664	3440	314	548	201	304	1367	4807
	2	1828	1811	3639	341	595	196	311	1443	5082
	3	1411	1336	2747	252	479	148	254	1133	3880
Early Adult	1	2365	2330	4695	376	665	234	415	1690	6385
	2	2639	2499	5138	459	788	296	461	2004	7142
	3	2415	2248	4663	406	715	255	414	1790	6453
Mid Adult	1	1582	1543	3125	256	449	153	264	1122	4247
	2	1495	1365	2860	245	417	129	238	1029	3889
	3	1575	1564	3139	271	469	164	277	1181	4320
Late Adult	1	2697	2593	5290	275	564	201	296	1336	6626
	2	2769	2696	5465	305	580	201	329	1415	6880
	3	2881	2840	5721	298	566	216	326	1406	7127

Table S2: SNP annotation at different stages of *Kerria lacca*

Sample (Stages)	Replication	Upstream	Exon	Intron	Downstream	Intergenic
Larvae	1	153	2137	702	559	1229
	2	157	2166	764	591	1377
	3	126	1608	640	458	1032
Early Adult	1	207	2807	1040	701	1596
	2	255	3101	1172	757	1821
	3	214	2834	1058	701	1619
Mid Adult	1	149	1845	679	502	1050
	2	125	1604	660	457	1025
	3	163	1670	775	480	1209
Late Adult	1	232	3236	848	690	1588
	2	249	3115	1009	737	1739
	3	247	3400	1026	784	1647

Table S3: InDel annotation at different stages of *Kerria lacca*

Sample (Stages)	Replication	Upstream	Exon	Intron	Downstream	Intergenic
Larvae	1	82	647	438	294	523
	2	87	694	437	342	554
	3	78	608	417	286	545
Early Adult	1	78	590	393	284	476
	2	82	613	415	295	479
	3	71	610	402	292	466
Mid Adult	1	89	579	400	284	429
	2	83	606	384	297	404
	3	79	605	435	304	511
Late Adult	1	83	587	307	253	396
	2	97	577	371	289	461
	3	89	605	339	269	421

Table S4: Genes associated with SNP variants

#Chr	Pos	Ref	Alt	Qual	Type	Gene
chr5	23746789	G	C	254.75	intergenic	gene_Kerria_yunnanensis0045760.1(dist=10185),gene_Kerria_yunnanensis0045770.1(dist=3388)
chr5	23753533	T	C	648.91	intronic	gene_Kerria_yunnanensis0045770.1
chr5	23753554	T	C	36.1	intronic	gene_Kerria_yunnanensis0045770.1
chr5	23753563	T	C	332.26	intronic	gene_Kerria_yunnanensis0045770.1
chr5	23753570	T	C	705.71	intronic	gene_Kerria_yunnanensis0045770.1
chr5	23776791	A	G	148.93	intergenic	gene_Kerria_yunnanensis0045770.1(dist=16841),gene_Kerria_yunnanensis0045780.1(dist=16240)
chr5	23776904	T	C	183.7	intergenic	gene_Kerria_yunnanensis0045770.1(dist=16954),gene_Kerria_yunnanensis0045780.1(dist=16127)
chr5	23777261	T	C	481.21	intergenic	gene_Kerria_yunnanensis0045770.1(dist=17311),gene_Kerria_yunnanensis0045780.1(dist=15770)
chr5	23778720	G	A	491.15	intergenic	gene_Kerria_yunnanensis0045770.1(dist=18770),gene_Kerria_yunnanensis0045780.1(dist=14311)
chr5	23779082	A	G	10372.6	intergenic	gene_Kerria_yunnanensis0045770.1(dist=19132),gene_Kerria_yunnanensis0045780.1(dist=13949)
chr5	23779084	A	G	1880.46	intergenic	gene_Kerria_yunnanensis0045770.1(dist=19134),gene_Kerria_yunnanensis0045780.1(dist=13947)
chr4	20041782	C	T	15372.5	intergenic	gene_Kerria_yunnanensis0054460.1(dist=21033),gene_Kerria_yunnanensis0054470.1(dist=32084)
chr3	13411115	C	T	392.04	exonic	gene_Kerria_yunnanensis0017940.1
chr3	13440194	C	T	107.99	intergenic	gene_Kerria_yunnanensis0017940.1(dist=17205),gene_Kerria_yunnanensis0017950.1(dist=1720)
chr1	7309099	C	T	77.83	downstream	gene_Kerria_yunnanensis0002510.1(dist=825)
chr3	18260212	A	G	14316	exonic	gene_Kerria_yunnanensis0019660.1
chr5	17276003	A	C	374.33	intronic	gene_Kerria_yunnanensis0043530.1
chr9	19187642	G	A	2818.56	UTR5	gene_Kerria_yunnanensis0063370.1(Kerria_yunnanensis0063370.1:c.-564G>A)
chr1	1539040	G	A	236.08	splicing	gene_Kerria_yunnanensis0000530.1(Kerria_yunnanensis0000530.1:exon7:c.943-1G>A)
chr5	16368991	A	G	553.67	UTR3	gene_Kerria_yunnanensis0043050.1(Kerria_yunnanensis0043050.1:c.*250T>C)
chr5	16371880	G	A	236332	exonic	gene_Kerria_yunnanensis0043050.1
chr5	16373190	A	G	199.23	upstream	gene_Kerria_yunnanensis0043050.1(dist=602)
chr5	16373193	G	A	198.77	upstream	gene_Kerria_yunnanensis0043050.1(dist=605)
chr5	16373332	G	A	167.29	upstream	gene_Kerria_yunnanensis0043050.1(dist=744)
chr5	16374269	G	A	407.49	intergenic	gene_Kerria_yunnanensis0043050.1(dist=1681),gene_Kerria_yunnanensis0043060.1(dist=1780)
chr5	16374308	A	G	297.39	intergenic	gene_Kerria_yunnanensis0043050.1(dist=1720),gene_Kerria_yunnanensis0043060.1(dist=1741)
chr1	5927857	A	T	344.22	intergenic	gene_Kerria_yunnanensis0001970.1(dist=32338),gene_Kerria_yunnanensis0001980.1(dist=68938)
chr1	5927859	G	A	73.13	intergenic	gene_Kerria_yunnanensis0001970.1(dist=32340),gene_Kerria_yunnanensis0001980.1(dist=68936)
chr1	10273522	T	C	4978.03	UTR3	gene_Kerria_yunnanensis0003520.1(Kerria_yunnanensis0003520.1:c.*329A>G)
chr1	10273523	T	C	507.65	UTR3	gene_Kerria_yunnanensis0003520.1(Kerria_yunnanensis0003520.1:c.*328A>G)
chr1	10273669	T	C	1205.5	UTR3	gene_Kerria_yunnanensis0003520.1(Kerria_yunnanensis0003520.1:c.*182A>G)
chr5	24577069	T	G	183.49	intronic	gene_Kerria_yunnanensis0046200.1

chr2	11056916	T	C	17404.5	exonic	gene_Kerria_yunnanensis0071240.1
chr2	11058198	G	A	1392.52	exonic	gene_Kerria_yunnanensis0071240.1
chr2	11058207	G	A	7339.22	exonic	gene_Kerria_yunnanensis0071240.1
chr2	11058345	T	A	14350.1	exonic	gene_Kerria_yunnanensis0071240.1
chr2	11058801	A	G	9660.6	exonic	gene_Kerria_yunnanensis0071240.1
chr2	11059895	G	A	4597	exonic	gene_Kerria_yunnanensis0071240.1
chr8	7625995	C	T	77.99	intronic	gene_Kerria_yunnanensis0028390.1
chr8	7628480	G	A	1641.39	exonic	gene_Kerria_yunnanensis0028390.1
chr8	7634729	C	T	2668.15	exonic	gene_Kerria_yunnanensis0028390.1
chr8	7636072	T	C	4286.99	exonic	gene_Kerria_yunnanensis0028390.1
chr1	20949510	G	A	197.16	intergenic	gene_Kerria_yunnanensis0008070.1(dist=3749),gene_Kerria_yunnanensis0008080.1(dist=1254)
chr1	20951341	G	A	1610.83	intronic	gene_Kerria_yunnanensis0008080.1
chr3	24320204	A	G	3332.08	intergenic	gene_Kerria_yunnanensis0022330.1(dist=2876),gene_Kerria_yunnanensis0022340.1(dist=2550)
chr3	24322695	A	G	1342	downstream	gene_Kerria_yunnanensis0022340.1(dist=59)
chr3	24325438	A	G	17609.4	upstream	gene_Kerria_yunnanensis0022340.1(dist=887)
chr5	20356846	T	G	2369.09	intronic	gene_Kerria_yunnanensis0044650.1
chr5	20356847	T	G	74.44	intronic	gene_Kerria_yunnanensis0044650.1
chr8	9162156	A	G	406.36	downstream	gene_Kerria_yunnanensis0028680.1,gene_Kerria_yunnanensis0028690.1(dist=181)
chr8	9162745	C	T	477.88	UTR3	gene_Kerria_yunnanensis0028690.1(Kerria_yunnanensis0028690.1:c.*478G>A)
chr8	9165374	T	C	4464.76	exonic	gene_Kerria_yunnanensis0028690.1
chr8	9165430	T	C	3502.8	exonic	gene_Kerria_yunnanensis0028690.1
chr8	9168943	G	A	277.38	upstream	gene_Kerria_yunnanensis0028690.1,gene_Kerria_yunnanensis0028700.1(dist=619)
chr5	16849914	A	G	7941.77	UTR3	gene_Kerria_yunnanensis0043180.1(Kerria_yunnanensis0043180.1:c.*389T>C)
chr5	24317578	A	G	739.1	exonic	gene_Kerria_yunnanensis0046050.1
chr3	13333518	C	T	1842.33	intergenic	gene_Kerria_yunnanensis0017920.1(dist=44714),gene_Kerria_yunnanensis0017930.1(dist=63540)
chr3	13357300	C	A	756.6	intergenic	gene_Kerria_yunnanensis0017920.1(dist=68496),gene_Kerria_yunnanensis0017930.1(dist=39758)
chr3	13357447	C	T	1197.81	intergenic	gene_Kerria_yunnanensis0017920.1(dist=68643),gene_Kerria_yunnanensis0017930.1(dist=39611)
chr1	1250642	A	C	6274.36	downstream	gene_Kerria_yunnanensis0000420.1(dist=417)
chr1	1250643	A	C	910.44	downstream	gene_Kerria_yunnanensis0000420.1(dist=416)
chr3	18310059	C	T	1341.23	exonic	gene_Kerria_yunnanensis0019680.1
chr3	18313603	T	C	1447.19	UTR3	gene_Kerria_yunnanensis0019680.1(Kerria_yunnanensis0019680.1:c.*329T>C)
chr3	18317631	C	A	1617.57	intergenic	gene_Kerria_yunnanensis0019680.1(dist=3275),gene_Kerria_yunnanensis0019690.1(dist=5280)
chr3	18317768	A	G	431.92	intergenic	gene_Kerria_yunnanensis0019680.1(dist=3412),gene_Kerria_yunnanensis0019690.1(dist=5143)
chr3	18317795	T	G	295.96	intergenic	gene_Kerria_yunnanensis0019680.1(dist=3439),gene_Kerria_yunnanensis0019690.1(dist=5116)

chr3	18317885	A	G	662.16	intergenic	gene_Kerria_yunnanensis0019680.1(dist=3529),gene_Kerria_yunnanensis0019690.1(dist=5026)
chr3	18318131	A	T	107.31	intergenic	gene_Kerria_yunnanensis0019680.1(dist=3775),gene_Kerria_yunnanensis0019690.1(dist=4780)
chr3	18318396	C	T	1609.7	intergenic	gene_Kerria_yunnanensis0019680.1(dist=4040),gene_Kerria_yunnanensis0019690.1(dist=4515)
chr3	18318555	G	A	1006.01	intergenic	gene_Kerria_yunnanensis0019680.1(dist=4199),gene_Kerria_yunnanensis0019690.1(dist=4356)
chr3	18319418	C	A	810.42	intergenic	gene_Kerria_yunnanensis0019680.1(dist=5062),gene_Kerria_yunnanensis0019690.1(dist=3493)
chr3	18319434	T	A	826.58	intergenic	gene_Kerria_yunnanensis0019680.1(dist=5078),gene_Kerria_yunnanensis0019690.1(dist=3477)
chr3	18319683	T	C	3158.17	intergenic	gene_Kerria_yunnanensis0019680.1(dist=5327),gene_Kerria_yunnanensis0019690.1(dist=3228)
chr3	18320583	T	A	369.41	intergenic	gene_Kerria_yunnanensis0019680.1(dist=6227),gene_Kerria_yunnanensis0019690.1(dist=2328)

Table S5: Genes associated with InDel variants

#Chr	Pos	Ref	Alt	Qual	Length	Type	Gene
chr5	23775786	T	TA	92.14	1	intergenic	gene_Kerria_yunnanensis0045770.1(dist=15836),gene_Kerria_yunnanensis0045780.1(dist=17245)
chr5	23752224	A	AT	86.62	1	intronic	gene_Kerria_yunnanensis0045770.1
chr1	7310856	T	TA	128.52	1	intronic	gene_Kerria_yunnanensis0002510.1
chr4	20028498	A	AT	193.43	1	intergenic	gene_Kerria_yunnanensis0054460.1(dist=7749),gene_Kerria_yunnanensis0054470.1(dist=45368)
chr4	20044728	C	CA	138.26	1	intergenic	gene_Kerria_yunnanensis0054460.1(dist=23979),gene_Kerria_yunnanensis0054470.1(dist=29138)
chr4	20044908	G	GA	264.51	1	intergenic	gene_Kerria_yunnanensis0054460.1(dist=24159),gene_Kerria_yunnanensis0054470.1(dist=28958)
chr4	20020665	G	GA	71.2	1	UTR3	gene_Kerria_yunnanensis0054460.1(Kerria_yunnanensis0054460.1:c.*149_*150insA)
chr4	20020763	A	AT	153.94	1	downstream	gene_Kerria_yunnanensis0054460.1(dist=14)
chr5	17276000	A	AC	60.06	1	intronic	gene_Kerria_yunnanensis0043530.1
chr5	17291939	C	CAAGAA	569.25	5	intergenic	gene_Kerria_yunnanensis0043530.1(dist=11976),gene_Kerria_yunnanensis0043540.1(dist=16844)
chr5	17291943	A	AAAAG	3299.97	4	intergenic	gene_Kerria_yunnanensis0043530.1(dist=11980),gene_Kerria_yunnanensis0043540.1(dist=16840)
chr5	17291945	A	AAG	134.38	2	intergenic	gene_Kerria_yunnanensis0043530.1(dist=11982),gene_Kerria_yunnanensis0043540.1(dist=16838)
chr1	1536982	C	CT	103.6	1	intronic	gene_Kerria_yunnanensis0000530.1
chr3	4660084	C	CA	280.57	1	intergenic	gene_Kerria_yunnanensis0013800.1(dist=1296),gene_Kerria_yunnanensis0013810.1(dist=1036)
chr1	10273515	T	TC	8261.39	1	UTR3	gene_Kerria_yunnanensis0003520.1(Kerria_yunnanensis0003520.1:c.*335_*336insG)
chr1	6543338	C	CT	86.56	1	intronic	gene_Kerria_yunnanensis0002170.1
chr1	6543345	G	GT	333.07	1	intronic	gene_Kerria_yunnanensis0002170.1
chr1	6543576	C	CA	168.15	1	intronic	gene_Kerria_yunnanensis0002170.1
chr5	24588931	T	TA	135.2	1	intronic	gene_Kerria_yunnanensis0046210.1
chr1	20946897	T	TA	134.54	1	intergenic	gene_Kerria_yunnanensis0008070.1(dist=1136),gene_Kerria_yunnanensis0008080.1(dist=3867)
chr1	20949051	G	GA	456.98	1	intergenic	gene_Kerria_yunnanensis0008070.1(dist=3290),gene_Kerria_yunnanensis0008080.1(dist=1713)
chr1	20949090	A	AT	126.16	1	intergenic	gene_Kerria_yunnanensis0008070.1(dist=3329),gene_Kerria_yunnanensis0008080.1(dist=1674)
chr3	24343624	C	CA	158.86	1	intergenic	gene_Kerria_yunnanensis0022350.1(dist=5961),gene_Kerria_yunnanensis0022360.1(dist=20756)
chr5	20356845	T	TG	381.92	1	intronic	gene_Kerria_yunnanensis0044650.1
chr5	20360950	A	AT	223.43	1	downstream	gene_Kerria_yunnanensis0044650.1(dist=806)
chr5	16858160	T	TTTC	3104.74	3	intronic	gene_Kerria_yunnanensis0043180.1
chr5	16858161	T	TTC	622.25	2	intronic	gene_Kerria_yunnanensis0043180.1
chr1	33062380	T	TA	100.02	1	intergenic	gene_Kerria_yunnanensis0012320.1(dist=5417),gene_Kerria_yunnanensis0012330.1(dist=4783)
chr1	1249889	G	GA	298.44	1	intergenic	gene_Kerria_yunnanensis0000410.1(dist=51190),gene_Kerria_yunnanensis0000420.1(dist=1170)
chr1	1250008	G	GA	125.14	1	intergenic	gene_Kerria_yunnanensis0000410.1(dist=51309),gene_Kerria_yunnanensis0000420.1(dist=1051)
chr1	1259354	A	AT	169.45	1	intronic	gene_Kerria_yunnanensis0000420.1
chr1	1250464	A	AAAAAG	203.24	5	downstream	gene_Kerria_yunnanensis0000420.1(dist=595)

chr1	1250465	A	AAAAG	1464.37	4	downstream	gene_Kerria_yunnanensis0000420.1(dist=594)
chr1	1250466	A	AAAG	2254.2	3	downstream	gene_Kerria_yunnanensis0000420.1(dist=593)
chr1	1250467	A	AAG	197.28	2	downstream	gene_Kerria_yunnanensis0000420.1(dist=592)
chr1	1250641	A	AC	779.13	1	downstream	gene_Kerria_yunnanensis0000420.1(dist=418)
chr1	1251006	T	TA	2190.62	1	downstream	gene_Kerria_yunnanensis0000420.1(dist=53)
chr4	22776443	C	CT	7794.8	1	intronic	gene_Kerria_yunnanensis0055150.1
chr4	22776684	G	GT	200.81	1	intronic	gene_Kerria_yunnanensis0055150.1
chr4	16676489	A	AT	5595.15	1	downstream	gene_Kerria_yunnanensis0053140.1,gene_Kerria_yunnanensis0053150.1(dist=421)
